# Supplementary material for: Community-led change: Progress toward policy, systems, and environmental impacts through the Catalyzing Communities initiative
Source: PLoS One. 2025 Nov 10;20(11):e0336482. doi: 10.1371/journal.pone.0336482 (PMC12599966; doi:10.1371/journal.pone.0336482)
Supplement: S1 File — (DOCX) [file pone.0336482.s001.docx]

## **Supplemental Material 1**

**Article Title:** Community-Led Change: Progress Toward Policy, Practice, and Environmental Impacts through the Catalyzing Communities Initiative

**Journal Name:** Journal of Community Health

**Author Names:** Travis R. Moore, Yuilyn A. Chang Chusan, Emily Sanderson, Larissa Calancie, Erin Hennessy, Julie Appel, Mary Ulseth, Christina D. Economos

**Affiliation and E-mail Address of Corresponding Author:** Travis R. Moore, ChildObesity180, Friedman School of Nutrition Science and Policy, Tufts University, Boston, MA; [Travis.Moore@Tufts.edu](mailto:Travis.Moore@Tufts.edu)

## **Interview Protocol**

The interview protocol included the following questions:

1. What significant relationships did you and/or the stakeholder committee form because of our work together?
2. What contextual factors within your organization, the stakeholder committee, or your community may have influenced the committee’s progress toward policy, systems, and/or environmental change?
3. How has partnering with Catalyzing Communities changed your approach to your own work?
4. Has partnering with Catalyzing Communities influenced your approach to promoting equity in your community?
5. Were there any measurable indicators or milestones that helped the stakeholder committee gauge progress?
6. What, if any, funding opportunities (secured or not secured) resulted from partnering with Catalyzing Communities during Phase I of the intervention?
7. What communication materials and/or dissemination products came out of partnering with Catalyzing Communities during Phase I of the intervention?
