# Supplementary material for: Community-led change: Progress toward policy, systems, and environmental impacts through the Catalyzing Communities initiative
Source: PLoS One. 2025 Nov 10;20(11):e0336482. doi: 10.1371/journal.pone.0336482 (PMC12599966; doi:10.1371/journal.pone.0336482)
Supplement: S2 File — (DOCX) [file pone.0336482.s002.docx]

## **Supplemental Material 2**

**Article Title:** Community-Led Change: Progress Toward Policy, Practice, and Environmental Impacts through the Catalyzing Communities Initiative

**Journal Name:** Journal of Community Health

**Author Names:** Travis R. Moore, Yuilyn A. Chang Chusan, Emily Sanderson, Larissa Calancie, Erin Hennessy, Julie Appel, Mary Ulseth, Christina D. Economos

**Affiliation and E-mail Address of Corresponding Author:** Travis R. Moore, ChildObesity180, Friedman School of Nutrition Science and Policy, Tufts University, Boston, MA; [Travis.Moore@Tufts.edu](mailto:Travis.Moore@Tufts.edu)

## **Interviewers**

The interviews and focus groups were conducted by five researchers: TM (PhD, postdoctoral scholar, male, with formal training in conducting interviews and over a decade of experience), LC (PhD, assistant professor, female, with formal training in conducting interviews and over a decade of experience), YC (PhD Candidate, female, with formal training and experience in conducting interviews), MU (Project Coordinator, female, with formal training and experience in conducting interviews), and JA (MS, senior project manager, female, with over five years of experience in conducting interviews). Participants were informed about the general goals of the research project and the interviewers' reasons and interests in the research.
