# Supplementary material for: Community-led change: Progress toward policy, systems, and environmental impacts through the Catalyzing Communities initiative
Source: PLoS One. 2025 Nov 10;20(11):e0336482. doi: 10.1371/journal.pone.0336482 (PMC12599966; doi:10.1371/journal.pone.0336482)
Supplement: S3 File — (DOCX) [file pone.0336482.s003.docx]

## **Supplemental Material 3**

**Article Title:** Community-Led Change: Progress Toward Policy, Practice, and Environmental Impacts through the Catalyzing Communities Initiative

**Journal Name:** Journal of Community Health

**Author Names:** Travis R. Moore, Yuilyn A. Chang Chusan, Emily Sanderson, Larissa Calancie, Erin Hennessy, Julie Appel, Mary Ulseth, Christina D. Economos

**Affiliation and E-mail Address of Corresponding Author:** Travis R. Moore, ChildObesity180, Friedman School of Nutrition Science and Policy, Tufts University, Boston, MA; [Travis.Moore@Tufts.edu](mailto:Travis.Moore@Tufts.edu)

## **Survey Questions**

1. What best describes your current involvement with Catalyzing Communities?
2. Please identify the initial programs, initiatives, or strategies* that you took or plan to make progress on initial those priority areas. (Please select all that apply. If there are any other action steps not listed, please provide them in the open text box.)
   Note: When we say "initial programs, initiatives, or strategies" we mean discrete activities (programs or initiatives) that the stakeholders chose to spend seed funding on.
3. Since your participation in GMB, has your organization or the stakeholder group decided to focus on different priority areas as a result of partnering with Catalyzing Communities?
4. Briefly describe how the stakeholder group's current priority areas have changed.
5. Please list any programs, initiatives, or strategies the stakeholder group is working on to make progress in these priority areas.
6. Please answer the following questions for each of the actions/strategies you selected earlier in the survey.
   1. To date, how much impact do you think this action had on achieving PSE change in your community
   2. What is the perceived level of completion for this action?
   3. How would you describe this action?
7. How successful was this action in reaching its target population?
