# Supplementary material for: Community-led change: Progress toward policy, systems, and environmental impacts through the Catalyzing Communities initiative
Source: PLoS One. 2025 Nov 10;20(11):e0336482. doi: 10.1371/journal.pone.0336482 (PMC12599966; doi:10.1371/journal.pone.0336482)
Supplement: S4 File — (DOCX) [file pone.0336482.s004.docx]

## **Supplemental Material 4**

**Article Title:** Community-Led Change: Progress Toward Policy, Practice, and Environmental Impacts through the Catalyzing Communities Initiative

**Journal Name:** Journal of Community Health

**Author Names:** Travis R. Moore, Yuilyn A. Chang Chusan, Emily Sanderson, Larissa Calancie, Erin Hennessy, Julie Appel, Mary Ulseth, Christina D. Economos

**Affiliation and E-mail Address of Corresponding Author:** Travis R. Moore, ChildObesity180, Friedman School of Nutrition Science and Policy, Tufts University, Boston, MA; [Travis.Moore@Tufts.edu](mailto:Travis.Moore@Tufts.edu)

## **Codebook**

| **Code** | **Definition** | **Supporting Evidence** |
| --- | --- | --- |
| Capacity building strategies that were offered as part of CC |  |  |
| Training | Pre-planned educational and/or skill-building sessions typically provided within group settings. | Leeman, Calancie et al 2015, adapted from EBSIS (Wandersman, 2012) |
| Tools | Informational and collaborative resources offered by Catalyzing Communities and designed to organize, summarize, and/or communicate knowledge. |  |
| Technical assistance | Interactive support that is individualized to the specific needs of individuals or teams. Those who provide TA may also be referred to as knowledge brokers, purveyors, linking agents, and external change agents among other terms. May also refer to the way in which meetings or support were facilitated by CC e.g., intentionally including and/or elevating diverse voices within the committee. |  |
| Assessment and feedback | Monitoring, providing or receiving feedback between CC and committee members related to the committee actions’ planning, adoption, implementation or dissemination |  |
| Peer networking | Bringing committee members and other stakeholders together to learn from each other via in-person or web-based training, TA, calls, discussions, or Learning Collaboratives. Note: This code focuses on CC’s act of bringing people together. The strategies conducted or the purpose of getting together may apply to other codes. Therefore, when applicable, peer networking may be double-coded. |  |
| Incentives | Incentives to motivate community partners to participate in the capacity-building intervention or to adopt and implement EBIs, such as scholarships to participate in training, or provision of free resources (e.g., stipends, seed funding, transportation, childcare vouchers) |  |
| SDCD constructs | Double code for anything that stemmed from the above SDCD activities |  |
| Knowledge | Stakeholders’ perceived understanding of various factors related to child healthy weights or related topic, including (i) the problem related to the topic, (ii) modifiable intervention factors, (iii) their own role and the role of others, (iv) how to intervene sustainably, and (v) available resources. Note: Not every time that words such as ‘knowledge’ or ‘knowing’ show up, will apply to this code. | Korn, Hennessy, Hammond et al 2018 |
| Engagement | Engagement was defined as a latent construct broadly representing stakeholders’ enthusiasm and agency for addressing child healthy weights or related topic.  Engagement includes the following domains: (i) bi-directional dialogue and mutual learning, (ii) flexibility, (iii) influence and power, (iv) leadership and stewardship, and (v) trust. |  |
| New social connections | Mention of newly established or strengthened social and/or professional relationships (i.e., connections between individuals that can serve a variety of purposes including social support, giving or receiving information, providing a sense of belonging) that occurred because of or during this project | Holt-Lunstad & Uchino, 2015 |
| Systems thinking | Approach to understanding complex phenomena by examining the interactions and interrelationships among various components within a system. It considers the system as a whole rather than focusing solely on its individual parts. | Meadows, 2008 |
| Diffusion | Sharing information, ideas, norms, enthusiasm, distrust, etc. through social and/or professional connections or networks. Note: mentions the spread. |  |
| Impacts |  |  |
| Funding or other material resources | Tangible or material resources, also referred to as instrumental, tangible or financial support that were at least partially attributed to the CC intervention | Holt-Lunstad & Uchino, 2015 |
| Credibility or status | Perceptions that those involved in the CC intervention, including participants, changemakers, and groups, have gained credibility or status in their communities | Calancie, Frerichs et al 2021 |
| Community empowerment | Perceptions that those involved in the CC were empowered or gained confidence to become more involved in changing conditions, practices, or relationships in their communities to create change. Note: Community empowerment focuses mostly on collective empowerment. | Calancie, Frerichs et al 2021; Empowerment Theory Fawcett et al. 1995 |
| Policy-related progress | Any measurable indicators, milestones, or descriptions of activities that create the conditions for problem identification, problem understanding, or policy development, adoption, implementation or evaluation that were at least partially attributable to the CC intervention | [Blanck, 2012](https://www.sciencedirect.com/science/article/abs/pii/S0749379712003959) |
| Behaviors | Descriptions of health-related behavior change at the individual level that could be at least partially attributable | Glass 2006 |
| Environments | Any measurable indicators, milestones, or descriptions of health-related environmental change in the physical or social spaces in which people live, work, or play that could be at least partially attributable to the CC intervention | Story 2008; Fleischhacker 2019; Illinois Prevention Research Center 2018 |
| Practices | A general category of activities, strategies, procedures or behaviors at the social, committee, or organizational levels that could be at least partially attributable to the CC intervention. Note: Programs may incorporate various practices. | [US Dept of Justice](https://crimesolutions.ojp.gov/about/comparing-programs-and-practices#:~:text=A%20program%20profile%20can%20answer,and%20how%20they%20address%20them.), [Nadia et al. 2017](file:///C:\Users\ychang08\AppData\Local\Box\Box%20Edit\Documents\oJNfFAiltkSeAHjDf1RVbg==\10.24095\hpcdp.37.11.03) |
| Health outcomes | Changes in physical, mental, well-being, disease or infirmity that are at least partially attributable to the CC intervention | WHO |
| Communication materials | Descriptions of materials or dissemination products, generic or tailored, that came out of partnering with the CC intervention (These materials do not have to be the ones we developed together) | Kreuter, Strecher & Glassman 1999 |
| Self-Efficacy | Stakeholder’s belief in their own ability to succeed in changing health-related behaviors, environments, or policies (individual, cognitive factor) | Flaspohler, Wandersman et al 2008 |
| Social justice | Description of more equitable distribution of power, resources, opportunities, and conditions for health and wellness, regardless of identity or social position. Note: Health equity is embedded within social justice. This may also refer to intentionally including and/or elevating diverse voices of external stakeholders. | Braveman & Gruskin 2003; Peter 2001 |
| Other |  |  |
| Contextual Factors | Contextual factors that influence progress toward policy, environmental, systems change such as setting capacity, evidence-based strategy attributes, stage of planning process beyond the CC intervention | Leeman, Calancie, et al 2015 |
| Amazing quote | A quote that really stands out and should be highlighted | Grounded theory; Charmaz, 2014 |
| Negative perceptions or places for improvement | Any quotes that indicate problems, unmet expectations, harm, or places for improvement in the CC approach |  |
| Self-growth | (if related to career growth) Career growth captures the results of one's efforts by defining it as one's perceptions of the chances of development and advancement within an organization. | [Weng, McElroy et al. 2020](https://www.sciencedirect.com/science/article/pii/S0001879110000850?casa_token=-80KE2-vvk8AAAAA:dyVXXwJ3hSV53A0POeq4oT71prm7QwYYE6oq_bZsWTSFRcs4vr7zIrM6FxLWayJni90WU95b#bib18) |
| Transformational learning | The critical reflection and reevaluate the assumptions they have made about themselves and their world | [Baumgartner 2001](https://d1wqtxts1xzle7.cloudfront.net/50938767/An_Update_on_Transformational_Learning20161217-28274-2ra3dx-libre.pdf?1482003016=&response-content-disposition=inline%3B+filename%3DAn_Update_on_Transformational_Learning.pdf&Expires=1711733469&Signature=OxLPT59XQVP~-Scy7zAUfz~Huc2C4QIQ0BORQwZsZX~LrFPJ7Gjx6WuB-IuUKfCTPgmq~239Q0CnzR0kzxv6HOa61LHPQO5nxjRKTHfzvuE3obBglEa5z~aWUjqy3FMhMK3wMFLQH-mccurm-PaeExMDkxA-cBz9YdmBc6~ZjAFXIfphOQzOLIMwh0jVbkCiryWHLhrH7fY2y9kUuWXnzz79AjOFOIWMde-GDEIqhmGbOHsv5b8hqbg7atfujcwVbYboa2Ua3hvZ0Hj9jCDIhxB1WQ90OGIOEsoO4BKH2bgBUfKxOjwLzaYb98SM9aAAnw1sCmzXTM~a68ZkKc5LtQ__&Key-Pair-Id=APKAJLOHF5GGSLRBV4ZA) |
| Feedback loop | To characterize non-linear relationships among constructs. Note: when coding for feedback loops, add a memo with the feedback loop connections. Ok to use the example quote shown here. | Grounded theory |
| Social-Ecological Model |  |  |
| Individual Factors | Knowledge, attitudes, behaviors, self-concept, skills, and developmental history of the individual | [McElroy et al 1988](https://journals.sagepub.com/doi/abs/10.1177/109019818801500401?casa_token=93FVU6Zc07AAAAAA:YdX3fqfPHcqKrCUzpTdY_VA78BvjGVGVsiljgPQgX5y7uBhDNbpPnquJ4BWy4YskRpZ4FTHwRB8&casa_token=O7PKOkqh3AYAAAAA:UL1X6ifAeIiGAH697VwGr4fS6FF97quy1S808Q6WxyNJl1AnMinzG-fC0rWgQ3jsBYJohNEdWoE) |
| Interpersonal Factors | Formal and informal social networks and social support systems (family, work group, friendships). May refer to internal or external relationships to the committee. For example, if it is mentioned that relationships were improved (but no explicit mention of who), you would use this code + new social connections. | [McElroy et al 1988](https://journals.sagepub.com/doi/abs/10.1177/109019818801500401?casa_token=93FVU6Zc07AAAAAA:YdX3fqfPHcqKrCUzpTdY_VA78BvjGVGVsiljgPQgX5y7uBhDNbpPnquJ4BWy4YskRpZ4FTHwRB8&casa_token=O7PKOkqh3AYAAAAA:UL1X6ifAeIiGAH697VwGr4fS6FF97quy1S808Q6WxyNJl1AnMinzG-fC0rWgQ3jsBYJohNEdWoE) |
| Interpersonal Factors (Committee) | Formal and informal social networks and social support systems that are specific to the internal relationships within the committee. This code is embedded within Interpersonal Factors. You can use this code to specify when relationships within the committee are mentioned explicitly. For example, if it is mentioned that committee members strengthened their relationship and supported each other, you would use this code + new social connections. | Grounded theory |
| Institutional | Social institutions defined as having organizational characteristics, formal rules, and regulations for operations | [McElroy et al 1988](https://journals.sagepub.com/doi/abs/10.1177/109019818801500401?casa_token=93FVU6Zc07AAAAAA:YdX3fqfPHcqKrCUzpTdY_VA78BvjGVGVsiljgPQgX5y7uBhDNbpPnquJ4BWy4YskRpZ4FTHwRB8&casa_token=O7PKOkqh3AYAAAAA:UL1X6ifAeIiGAH697VwGr4fS6FF97quy1S808Q6WxyNJl1AnMinzG-fC0rWgQ3jsBYJohNEdWoE) |
| Community | Relationships between organizations and institutions. Example: CBO or organizations relationships | [McElroy et al 1988](https://journals.sagepub.com/doi/abs/10.1177/109019818801500401?casa_token=93FVU6Zc07AAAAAA:YdX3fqfPHcqKrCUzpTdY_VA78BvjGVGVsiljgPQgX5y7uBhDNbpPnquJ4BWy4YskRpZ4FTHwRB8&casa_token=O7PKOkqh3AYAAAAA:UL1X6ifAeIiGAH697VwGr4fS6FF97quy1S808Q6WxyNJl1AnMinzG-fC0rWgQ3jsBYJohNEdWoE) |
| Policy | Local, state, national, or global laws and policies | [McElroy et al 1988](https://journals.sagepub.com/doi/abs/10.1177/109019818801500401?casa_token=93FVU6Zc07AAAAAA:YdX3fqfPHcqKrCUzpTdY_VA78BvjGVGVsiljgPQgX5y7uBhDNbpPnquJ4BWy4YskRpZ4FTHwRB8&casa_token=O7PKOkqh3AYAAAAA:UL1X6ifAeIiGAH697VwGr4fS6FF97quy1S808Q6WxyNJl1AnMinzG-fC0rWgQ3jsBYJohNEdWoE) |
